# Erchen Decoction Induces Browning Tendency in White Adipose Tissue of Obese Rats *via* the SP1/SREBP/UCP1 Signaling Pathway

**DOI:** 10.2174/0118715303415055251209122646

**Published:** 2026-06-23

**Authors:** Yue Luo, Mengzhen Wang, Donglin Liu, Fengyi Guo, Nan Song, Zhe Jin, Ziyu Liu, Xiao Yang

**Affiliations:** 1 Liaoning University of Traditional Chinese Medicine, Shenyang, China;; 2 The Second Affiliated Hospital of Liaoning University of Traditional Chinese Medicine, Shenyang, China;; 3 Liaoning Academy of Traditional Chinese Medicine Sciences, Shenyang, China;; 4 Shenyang Clinical Medical Research Center for Diabetic and Lipid Metabolic Diseases in Traditional Chinese Medicine, Shenyang, China;; 5 Shenyang Key Laboratory of Research and Transformation of New Traditional Chinese Medicine for Immune Inflammatory Diseases, Shenyang, China

**Keywords:** Erchen decoction, obesity, SP1/SREBP/UCP1, browning tendency, fat synthesis, white adipose tissue

## Abstract

**Objectives:**

Obesity is a global metabolic challenge that leads to excessive fat accumulation and adipose tissue dysfunction due to the disruption of the balance between energy intake and consumption. This study focuses on whether Erchen Decoction promotes high-fat diet-induced white fat browning in obese rats through the SP1/SREBP/UCP1 signaling pathway, thereby improving obesity.

**Methods:**

Male SD rats were fed a high-fat diet to establish an obesity model, and were randomly divided into the High-fat diet (HFD) group, Erchen Decoction low dose, medium dose, high dose groups, and high dose + SP1 inhibitor group. Rats fed a normal diet served as the control group. TC, TG, LDL-C and HDL-C were detected by an automatic biochemical analyzer; the morphology of adipose tissue was observed with HE staining; the expression of SP1, SREBP and UCP1 in adipose tissue was detected with immunofluorescence staining; the mRNA and protein expression levels of SP1, SREBP, UCP1, PPA γ, PGC-1α, p-p65, Fass and ACC in white adipose tissue were detected with Real-time PCR and Western blot respectively.

**Results:**

Each dose group of Erchen Decoction and the HD-SP1i group could effectively improve the body weight, blood lipids, and fat tissue morphology of obese rats. After the intervention of Erchen Decoction and HD-SP1i, the expressions of UCP1 and PGC-1α in rat adipose tissue cells were up-regulated, while the expressions of SP1, SREBP, PPA γ, p-p65, Fass, and ACC were inhibited.

**Discussion:**

Obesity is a significant global health challenge closely linked to metabolic disorders. According to Traditional Chinese Medicine (TCM) theory, it is often understood as stemming from spleen deficiency leading to the accumulation of phlegm-dampness. Erchen Decoction, a classic TCM formula, achieves its therapeutic effects through the synergistic action of its herbal components, which collectively strengthen the spleen, resolve phlegm, and eliminate dampness. This study demonstrates that Erchen Decoction effectively reduces body weight, improves dyslipidemia, and alleviates pathological changes in adipose tissue in obese rats. The findings indicate that the decoction promotes a "browning" tendency in white adipose tissue, as evidenced by the upregulation of thermogenic markers UCP1 and PGC-1α, and the downregulation of key lipogenic factors SP1, SREBP, Fas, and ACC. These observations suggest that the anti-obesity effects of Erchen Decoction may be mediated through the modulation of the SP1/SREBP/UCP1 signaling pathway, which induces white adipose tissue browning and suppresses lipogenesis. This study provides novel molecular insights into the anti-obesity mechanism of Erchen Decoction, enriching the evidence base for TCM in the treatment of metabolic diseases. Future research should aim to obtain direct histological evidence of UCP1 expression and the formation of multilocular adipocytes, and further explore the effects of the formula on related cellular stress pathways.

**Conclusion:**

Erchen Decoction may activate the SP1/SREBP/UCP1 signaling pathway and induce browning tendency in white adipose tissue, thereby exerting weight loss.

## 1 INTRODUCTION

Obesity is a chronic metabolic disorder characterized by an imbalance between energy intake and expenditure, with core pathological features including excessive total body fat accumulation, regional fat deposition, and/or abnormal fat distribution. As its global prevalence rises steadily year by year, obesity has evolved into a major public health concern of international attention [1]. Notably, China currently has the largest population of overweight or obese individuals worldwide [2]; epidemiological studies have shown that approximately 50% of adults and 20% of children in China are affected by overweight or obesity [3]. Beyond its impacts on physical appearance and quality of life, obesity significantly elevates the risk of developing multiple comorbid metabolic conditions, such as type 2 diabetes mellitus and cardiovascular diseases [4-6], thereby further amplifying its burden on public health. Therefore, implementing strategies to prevent obesity onset, enhancing public awareness of obesity-related hazards, and promoting healthy lifestyles are critical for mitigating the global obesity epidemic.

Functional and morphological remodeling of adipose tissue plays a pivotal role in the pathogenesis of obesity [7]. Adipose tissue is not only the primary site for energy storage but also functions as an endocrine organ that secretes a spectrum of bioactive molecules, including leptin, adiponectin, and resistin. These adipokines exert critical regulatory effects on energy metabolism, insulin sensitivity, and inflammatory responses [8]. Under obese conditions, adipose tissue undergoes profound pathophysiological alterations, such as adipocyte hypertrophy, increased adipocyte hyperplasia, and heightened adipose tissue inflammation. These aberrant changes further exacerbate the initiation and progression of obesity-associated metabolic disorders. In mammals, adipose tissue is primarily categorized into two functional subtypes: white adipose tissue (WAT) and brown adipose tissue (BAT). WAT primarily serves as an energy reserve, whereas BAT mediates the dissipation of chemical energy derived from nutrient oxidation as heat—an effect driven by uncoupling protein 1 (UCP1)—thereby contributing to the establishment of a negative energy balance [9, 10]. In recent years, the phenomenon of “white adipose tissue browning” (*i.e.*, the transformation of WAT into a BAT-like phenotype) has garnered extensive attention and is regarded as a promising therapeutic strategy for combating obesity and related metabolic disturbances [11-13]. Specifically, activating BAT or inducing WAT browning can enhance energy expenditure, promote weight reduction, and ameliorate metabolic dysfunction. Thus, in-depth investigations into the functional and morphological changes of adipose tissue—with a particular focus on the mechanisms underlying BAT activation and WAT browning—are of considerable importance for the development of novel therapeutic approaches targeting obesity.

Erchen Decoction is one of the classic traditional Chinese medicine prescriptions. According to Traditional Chinese Medicine (TCM) theory, this formula is believed to eliminate dampness and phlegm, regulate qi, and strengthen spleen function. It has been traditionally used in clinical practice for these purposes [14]. In recent years, the role of Erchen Decoction in improving obesity, regulating glycolipid metabolism, and enhancing insulin sensitivity has received increasing attention [15-18]. Existing studies have shown that Erchen Decoction can alleviate obesity-related liver steatosis by regulating the content of butyric acid produced by intestinal flora and promoting β-oxidation of fatty acids [19]. Other studies have shown that Erchen Decoction can regulate lipid metabolism by up-regulating the expression of PPAR γ and LPL, thereby effectively improving obesity and lipid metabolism disorders in mice induced by a high-fat diet [20]. However, the specific effect and mechanism of Erchen Decoction on the browning of white adipose tissue have not yet been fully clarified.

Specificity Protein 1 (SP1) is an important transcription factor that regulates the expression of target genes by interacting with a range of proteins, thereby helping to regulate multiple biological processes [21]. Uncoupling protein 1 (UCP1) is a protein unique to brown adipose tissue and is crucial for the thermogenic function of brown adipose tissue [22]. Sterol Regulatory Element-Binding Protein (SREBP) is a key transcription factor in cholesterol and fatty acid synthesis, and its activity is strictly regulated. Recent studies have confirmed that SP1 may participate in the regulation of lipid metabolism by regulating SREBP1 [23-25]. However, there is no clear report whether SP1 can regulate the expression of UCP1 through SREBP1 and thus affect the browning process of white adipose tissue. Therefore, this study aims to further explore and improve the mechanism of Erchen Decoction in promoting browning of white adipose tissue, investigate the regulatory effect of Erchen Decoction on the SP1/SREBP/UCP1 signaling pathway, and provide a scientific experimental basis for the development of drugs to prevent and treat obesity and related metabolic diseases.

## 2 MATERIALS AND METHODS

### 2.1 Animals

48 SPF healthy male Sprague-Dawley rats, weighing (220 ± 20) g, 8 weeks old, were purchased from Jiangsu Huachuang Xinnuo Pharmaceutical Technology Co., Ltd., with animal production license number SCXK (Su) 2020 - 0009. Before the experiment, all animals were placed indoors for 1 week to adapt to the environment. The feeding conditions were temperature (23 ± 2) °C and humidity (50 ± 15) %. Animals were allowed ad libitum access to standard rat feed and tap water. Both a high-fat diet (60% fat content: triglycerides) and a normal diet were purchased from Synergene Biotech Co., Inc.

### 2.2 Drug Preparation

The eight TCMs contained in Erchen Decoction (Table **1**) were purchased from the Affiliated Hospital of Liaoning University of Traditional Chinese Medicine and produced by Anhui Puren Traditional Chinese Medicine Slices Co., Ltd. The composition and sources of the drugs are as follows: Pinellia ternata 15 g, Citrus reticulata 'Blanco' 15 g, Wolfiporia extensa 9 g, honey-fried Glycyrrhiza uralensis 4.5 g, 7 slices of fresh *Zingiber officinale*, and 1 *Prunus mume*. All the above medicinal materials have been identified and meet the standards of the Chinese Pharmacopoeia (Edition 2020). Method of decocting the medicinal preparation: Immerse the medicinal materials in 460 mL of water for 1 hour, then bring to a boil over high heat and simmer over low heat for 1 hour. Add 460 mL of water and continue decocting for 30 minutes. Filter out the dregs with sterilized gauze, combine the filtrates, and concentrate to a final volume corresponding to 1.089 g of crude drug per mL. Store it at 4°C for future use.

### 2.3 Preparation of Obese Rat Models

Healthy male SD rats were randomly divided into the control group (Control group), High-fat diet group (HFD group), low-dose Erchen Decoction group (EC-L group), medium-dose Erchen Decoction group (EC-M group), high- dose Erchen Decoction group (EC-H group), and high-dose + SP inhibitor group (HD-SP1i group, Mithramycin A), with 8 rats in each group, totaling 6 groups. After being fed conventionally for 1 week, rats in the control group were fed a standard diet until the end of the experiment, while the rat model of obesity was induced by feeding the rats on a high- fat diet (HFD) (35% fat: triglycerides, 26% carbohydrate, and 26% protein) for 8 weeks. After 8 weeks, rats with body mass and body length exceeding those of the control group by 20% and with a multiple increase in Lee's index were selected as the criteria for successful modeling. Lee's index = × 10^3^ / nasal-anal length (cm).

### 2.4 Group Medication Measures

After successful animal modeling (HFD for 8 weeks), treatment was performed for 8weeks. The administration dose was calculated according to the clinical dosage for average adults (body weight: 70 kg) and the conversion formula for equivalent doses between animals and humans [19]. The rats in the EC-L, EC-M, and EC-H groups received ECD *via* oral gavage at daily doses of 0.5445, 1.089, and 2.178 g/kg, respectively, for 8 consecutive weeks, and the HD-SP1i group at daily doses of Erchen Decoction 2.178 g/kg and Mithramycin A 0.03 mg/kg each week (intraperitoneal injection) [26]. The control group and the HFD group were administered an equal volume of distilled water, once a day, for 8 consecutive weeks. The treatment was initiated in the 9^th^ week and concluded in the 16^th^ week. The body weight of each group of rats was measured every 2 weeks. At the end of the 16^th^ week, the animals were fasted overnight for 10 hours and then anesthetized with 40 mg/kg of 1% pentobarbital sodium by intraperitoneal injection for sample collection. Peripheral blood was collected *via* the posterior vena cava for subsequent experiments. The fat tissues were immediately removed and weighed.

### 2.5 Fully Automatic Biochemical Analyzer Detection

After extracting the serum from the blood of rats in each group, according to the instructions of the kit, a fully automatic biochemical analyzer (Chemray 240, Shenzhen Leadman Biochemistry Co., Ltd.) was used to detect the levels of total cholesterol (TC), triglyceride (TG), low-density lipoprotein (LDL-C) and high-density lipoprotein (HDL-C) in rat serum.

### 2.6 Hematoxylin and Eosin (HE) Staining

Take the adipose tissue fixed in 4% paraformaldehyde, perform gradient dehydration with xylene, embed in paraffin after dewaxing, and prepare 5 μm sections using an RM2016 pathological microtome (Leica, Shanghai). After dewaxing and rehydration of the paraffin sections, stain with hematoxylin for 5 min, differentiate with a differentiating solution, rinse with tap water, blue the sections after rinsing, perform gradient dehydration with alcohol again, stain with eosin for 5 min, dehydrate and transparentize the sections after staining, and finally seal the slides with neutral balsam. Observe the pathological changes in rat adipose tissue under a microscope and take photos. Image-Pro Plus 6.0 software (Media Cybernetics, Rockville, MD, USA) was used to measure adipocyte area, perimeter, and aspect ratio, as previously described [27].

### 2.7 Immunofluorescence Detection

After the tissue sections were placed at room temperature for 10 min, the paraffin sections were successively placed in xylene for 3 min, anhydrous ethanol for 5 min, 85% ethanol for 5 min, 75% ethanol for 5 min, and washed with ddH2O for 5 min for gradient dewaxing and hydration treatment. The sections were placed in a repair box filled with citrate antigen retrieval solution and subjected to antigen retrieval in a microwave oven. After the repair was completed, the sections were washed with PBS, and 5% BSA (Solebao A8010) was added dropwise in the circle and incubated for 30 min. After discarding the blocking solution, the primary antibodies UCP1 (Proteintech 23673-1-AP, 1:50), SP1 (Affinity AF6121, 1:80), and SREBP (Affinity AF6283, 1:100) were added dropwise. The sections were laid flat in a wet box and incubated overnight at 4°C. The next day, they were rewarmed at 37°C for 30 min, washed three times with PBS, and the corresponding secondary antibody (Beyotime A0562, 1:200) was added and incubated at 37°C in the dark for 1 h. After washing three times with PBS, DAPI was added for nuclear staining for 5 min. PBS was washed three more times, each time for 5 min. An anti-fluorescence quenching agent was used to seal the slides, and observations and photographs were taken under an inverted fluorescence microscope (OLYMPUS IX73). ImageJ software (Version 1.8.0) was used to measure the mean fluorescence intensity (MFI) of UCP1, SP1, and SREBP.

### 2.8 RT-qPCR

According to the instructions of TRIzol reagent (Solarbio R1100), after grinding the tissue samples, add 1 mL of Trizol and operate on ice. Vortex emulsify with chloroform (Guoyao Group Chemical Reagents Co., Ltd., 10006818) and centrifuge to separate. Take the supernatant, add isopropanol (Guoyao Group Chemical Reagents Co., Ltd., 40049962), mix well, let stand, and then centrifuge to obtain RNA precipitate. After washing with ethanol and centrifugation, dissolve the precipitate with RNA dissolving solution, and determine the RNA concentration by a spectrophotometer. Remove genomic DNA by using a reverse transcription kit. GAPDH served as the internal reference mRNA. Configure the reaction system on ice, incubate at 70°C for 5 min, and then immediately place on ice to cool. Centrifuge at 4°C for a short time. Add the reaction solution and reverse transcribe at 42°C for 60 min to synthesize cDNA (ToloScript RT EasyMix for qPCR (TOLOBIO 22106)). Use SYBR qPCR Master Mix (TOLOBIO 22204 - 01) for amplification. The reaction conditions are as follows: pre-denaturation at 95°C for 3 min, denaturation at 95°C for 30 s, annealing at 55°C for 20 s, 40 cycles, and extension at 72°C for 20 s. Quantitative analysis was performed by the 2-ΔΔCt method. The primers required for the experiment were synthesized and designed by Jiangsu Sesofei Biotechnology Co., Ltd (Table **2**).

### 2.9 Western Blot

The total white adipose tissue protein was extracted using RIPA lysis buffer (CWBIO, China) and quantified with a BCA protein determination kit (Beyotime, China). Heat the proteins at 95°C for 5 min to denature them. Load the samples onto the gel wells, with approximately 50 μg per well. Perform electrophoresis using the Tanon electrophoresis system with a constant current of 15 mA per gel. Stop electrophoresis when the target proteins are at the optimal resolution position, which is 1/3 down from the top of the separating gel. The transfer process is carried out at 4°C with 100 V/100 mA for 65 min to separate the target proteins and transfer them onto a PVDF membrane (Millipore IPVH00010). Block the PVDF membrane with 3% BSA for 2 h, and wash it three times with TBST, 10 min each time. Incubate the membrane overnight at 4°C with primary antibodies against PPARγ (Affinity AF6284, 1:800), p - p65 (Affinity AF2006, 1:1000), PGC - 1α (Proteintech 66369-1-Ig, 1:500), SP1 (Affinity AF6121, 1:500), SREBP (Affinity AF6283, 1:5000), UCP1 (Proteintech 23673-1-AP, 1:400), Fas (Affinity AF5342, 1:800), ACC (Affinity AF6421, 1:500), and β - actin (Affinity AF7018, 1:8000). Wash the membrane five times with TBST. Prepare the secondary antibody working solution at a dilution ratio of 1:1000 according to the instructions (Beyotime A0208, A0216). Incubate the membrane on a shaker at room temperature for 2 h. Wash the membrane five times with TBST, and then develop and expose it using an ECL working solution (Beyotime P0018S) on the Tanon-5200 imaging system. Save the bands and analyze the grayscale values with ImageJ.

### 2.10 Statistical Analysis

SPSS 26.0 software was used for statistical analysis of the data. If the normal distribution and homogeneity of variance conditions were met, one-way analysis of variance (one-way ANOVA) was subjected, followed by Tukey’s Honestly Significant Difference (HSD) test for post-hoc pairwise comparisons (to identify specific differences between groups). Results were expressed as mean ± standard deviation (mean ± SD). If the data failed to meet the normality assumption or the requirements of homogeneity of variance, non-parametric tests were used. *P* < 0.05 indicated a statistically significant difference. GraphPad Prism 8 software was used for drawing graphs.

## 3 RESULTS

### 3.1 The Effect of Erchen Decoction on Body Weight in Obese Rats

The experimental results showed that the body weight of the High-fat diet group (HFD) rats was significantly higher than that of the normal control group (Control) from the 2nd week, indicating successful modeling (Fig. **1**). Under the intervention of Erchen Decoction, the body weight of rats in each dose group of Erchen Decoction (EC-L, EC-M, EC-H) and the HD-SP1i group was significantly reduced. Two weeks after administration, the rate of weight loss in rats of each intervention group was increased, and after four weeks of administration, the weight loss effect was more significant; by eight weeks of administration, the weight loss of rats in each intervention group continued to decrease, indicating that Erchen Decoction has a significant weight loss effect, and with the extension of administration time (from 2 weeks to 4 weeks to 8 weeks), the weight loss effect of Erchen Decoction gradually increased.

### 3.2 Effect of Erchen Decoction on Blood Lipid Levels in Obese Rats

Compared with the normal control group, obese rats had obvious dyslipidemia (Fig. **2**). The serum TC, TG, and LDL-C levels in HFD rats were significantly increased, while the HDL-C level decreased (*P* < 0.01). After the intervention of each dose group of Erchen Decoction and the HD-SP1i group, the serum TC, TG, and LDL-C levels in rats were significantly decreased, and the HDL-C level increased (*P* < 0.01). It can be seen that Erchen Decoction can effectively improve the lipid metabolism of obese rats (Table **3**).

### 3.3 HE Staining was used to Observe the Morphological Changes of Fat Tissue in Obese Rats Treated with Erchen Decoction

The results of HE staining showed that the adipocytes in the normal control group (Control) were uniform in size and arranged regularly. In the High-fat diet group (HFD), the volume of adipocytes increased significantly, with irregular morphology. Fat vacuoles appeared in some areas, accompanied by cell swelling. After intervention with Erchen Decoction, the volume of adipocytes decreased, the number increased, and the arrangement became more compact in each dosage group and the HD-SP1i group. The degree of adipocyte lesions was significantly alleviated, indicating that Erchen Decoction can improve the pathological morphology of adipose tissue in obese rats (Fig. **3**).

### 3.4 Effect of Erchen Decoction on the Expression of UCP1, SP1 and SREBP in Adipose Tissue of Obese Rats

The results of immunofluorescence staining (Figs. **4**-**6**) showed that after the intervention of Erchen Decoction at each dose group and HD-SP1i group, the expression of UCP1 in adipose tissue of obese rats was significantly up- regulated, while the expression of SP1 and SREBP was suppressed (*P* < 0. 01), suggesting that Erchen Decoction may improve obesity by regulating the expression of UCP1, SP1 and SREBP.

### 3.5 Effects of Erchen Decoction on the mRNA and Protein Levels of SP1, SREBP, UCP1, PPARγ, PGC-1α, p-p65, Fas, and ACC in Adipose Tissue of Obese Rats

The results of RT-qPCR (Fig. **7**) and WB (Fig. **8**) showed that the mRNA and protein expression levels of UCP1 and PGC-1α were significantly decreased in adipose tissue of obese rats, while the expressions of SP1, SREBP, PPA γ, p-p65, Fass, and ACC were increased (*P* < 0.01 or *P* < 0.05). After intervention in various dose groups of Erchen Decoction and HD-SP1i group, the expressions of UCP1 and PGC-1α were significantly up-regulated, and the expressions of SP1, SREBP, PPA γ, p-p65, Fass and ACC were down-regulated at the same time (*P* < 0.01 or *P* < 0.05), suggesting that Erchen Decoction may improve lipid metabolism disorders in obese rats by regulating the expression of lipid metabolism related genes (UCP1, PGC-1α, SP1, SREBP, *etc*.).

## 4 DISCUSSION

Currently, obesity has become an increasingly severe global public health issue, with complex pathophysiological mechanisms involving multiple signaling pathways and metabolic processes [28-31]. It not only significantly impacts individual health but also imposes a heavy socioeconomic burden. At present, the treatment methods of obesity include lifestyle intervention, anti-obesity drugs, endoscopic treatment, and weight loss surgery. However, these methods have some disadvantages, such as long-term effects and safety to be evaluated, and individual responses are very different [32].

According to TCM theory, obesity is associated with patterns such as “phlegm-dampness” and “spleen dysfunction”. These traditional diagnostic categories may correspond to metabolic dysregulation, poor diet, and physical inactivity in biomedical terms. Modern lifestyles, characterized by excessive consumption of rich, fatty foods and reduced physical activity, lead to impaired spleen function, internal dampness accumulation, and phlegm-dampness retention, thereby contributing to obesity. When the spleen fails to transport and transform nutrients, the stomach may still receive food, but the ingested nutrients cannot be converted into essential substances for bodily functions. Instead, these stagnate internally, transforming into pathological products such as dampness, phlegm, and blood stasis, which accumulate in the body. Spleen deficiency, over time, generates turbid stagnation, further depleting spleen and stomach qi, creating a vicious cycle that reduces metabolic efficiency and accelerates obesity progression. Thus, “spleen qi deficiency and impaired spleen function” are key factors in the development and progression of obesity. By analyzing the pathogenesis of obesity and adhering to the principle of “treatment based on syndrome differentiation”, the therapeutic approach of “fortifying the spleen, resolving phlegm, and dispelling dampness” is employed, with Erchen Decoction serving as an effective treatment.

Erchen Decoction is a classic TCM formula. Pinellia ternata, as the sovereign herb, dries dampness and resolves phlegm; aged Decoctionerine peel regulates qi and aids phlegm resolution; Poria cocos strengthens the spleen and leaches out dampness, preventing its accumulation; licorice harmonizes the formula and moderates the middle burner. Together, these herbs synergistically fortify the spleen, resolve phlegm, dispel dampness, and reduce fat. Recent advances in modern pharmacological research have gradually elucidated the mechanisms by which Erchen Decoction and its modified formulations improve glucose and lipid metabolism disorders and aid in weight loss [17, 33]. Studies indicate that active components in Pinellia ternata can modulate the expression of genes related to lipid metabolism [34, 35], while flavonoids in aged Decoctionerine peel exhibit anti-inflammatory and antioxidant properties [36, 37]. Poria cocos polysaccharides regulate gut microbiota balance, further promoting fat metabolism [38, 39]. Additionally, glycyrrhizic acid and glycyrrhetinic acid in licorice show potential therapeutic effects on metabolic diseases [40, 41]. These findings suggest that the multi-component, multi-target mechanism of Erchen Decoction may underlie its efficacy in treating obesity.

As research on obesity-related diseases deepens, white adipose tissue browning has emerged as a critical pathway for regulating energy balance and improving metabolism. This process involves the conversion of white adipocytes into metabolically active brown-like adipocytes under specific stimuli. Brown fat achieves respiratory uncoupling through its high mitochondrial density and the expression of UCP1, which promotes the conversion of chemical energy to heat energy and reduces fat accumulation. Previous studies have demonstrated that Erchen Decoction, as a traditional herbal formula, exhibits potential therapeutic effects in reducing body weight, improving insulin resistance, and modulating lipid metabolism [17]. It interferes with the insulin receptor substrate 1 (IRS1)/protein kinase B (AKT)/protein kinase A (PKA)/hormone-sensitive lipase (HSL) signaling pathway, reducing the concentration of free fatty acids (FFAs) released due to excessive lipolysis in white adipose tissue [17]. In this study, we observed that Erchen Decoction significantly reduced body weight in obese rats while upregulating uncoupling protein 1 (UCP1) expression, suggesting that it may promote white adipose tissue browning, enhance energy expenditure, and improve metabolic function.

The SP1/SREBP/UCP1 signaling pathway is a key regulator of lipid metabolism and energy balance. SP1, as a transcription factor, modulates SREBP expression, influencing fatty acid synthesis and storage [42]. SP1 activation increases SREBP expression, thereby promoting the expression of key enzymes such as fatty acid synthase (FAS) and acetyl- CoA carboxylase (ACC), accelerating fat accumulation. Conversely, UCP1, a hallmark protein of brown adipose tissue, uncouples respiration, dissipating energy as heat and counteracting fat deposition [43]. This pathway plays a significant role in metabolic disorders such as obesity and diabetes, with dysregulation closely linked to disease progression. Recent studies have found that increased SP1 activity promotes SREBP expression [44, 45], and their synergistic activation may exacerbate fat synthesis and insulin resistance. Other research highlights that upregulating UCP1 expression effectively promotes white-to-brown fat transformation, alleviating obesity symptoms [46, 47]. Our findings align with these observations, indicating that Erchen Decoction may inhibit SP1 activity, thereby reducing SREBP expression and suppressing FAS and ACC activity, slowing fat accumulation. Simultaneously, it upregulates UCP1 expression, promoting energy expenditure, which may facilitate white adipose tissue browning and counteract obesity.

In this study, an obese rat model was established to investigate the effects of Erchen Decoction on the body weight of obese rats, morphological changes in adipose tissue, and the expression of signal molecules such as UCP1, SP1, and SREBP in adipose tissue. We found that in the obese rat model, SP1 and SREBP expression were upregulated, while UCP1 expression was downregulated, which is consistent with the pathological characteristics of obesity. After Erchen Decoction intervention, this abnormal regulation was significantly reversed, manifested as a decrease in SP1 and SREBP expression and an increase in UCP1 expression. Meanwhile, the upregulation of the white adipose browning marker gene PGC-1α and the downregulation of fat synthesis-related genes such as PPARγ, p-p65, Fas, and ACC were also observed. These results not only verify the effect of Erchen Decoction in inducing the browning tendency in white adipose tissue but also reveal that it may achieve this effect by regulating the SP1/SREBP/UCP1 signaling pathway, echoing the previous research reports on the effects of Erchen Decoction in improving metabolism and reducing weight. The above research results suggest that Erchen Decoction may promote the conversion of white adipose tissue into brown adipose tissue by regulating the SP1/SREBP/UCP1 signaling pathway, thereby inhibiting fat synthesis and alleviating obesity symptoms.

It is important to note that obesity-induced hyperlipidemia is not only a consequence of adipose tissue dysfunction but also an important risk factor for systemic complications, including atherosclerosis. Recent studies have found that 2-acetylaminophenol (2-AAP) also significantly reduces hyperlipidemia by regulating ferroptosis and glutathione metabolism, effectively inhibiting the progression of atherosclerosis [48]. A growing body of research evidence suggests that adipose tissue may be exposed to a variety of cellular damage threats, including oxidative stress and ferroptosis, under metabolic stress [49, 50]. In this study, Erchen Decoction effectively induced the browning tendency in white adipose tissue and improved the metabolic activity of the tissue. We speculated that Erchen Decoction may improve the overall fat metabolism homeostasis by regulating the SP1/SREBP/UCP1 pathway. Future studies will further explore the specific effects of Erchen Decoction on cell death pathways, such as ferroptosis, in obese adipose tissue to more fully reveal its multi-target mechanism for improving metabolism.

In addition, although this research proposed that Erchen Decoction induced the browning tendency in white adipose tissue through the SP1/SREBP/UCP1 signaling pathway, the specific mode of action of this pathway, the interaction between the components, and the cross-talk with other signaling pathways may not have been deeply discussed. Besides, this study has several limitations: results from WB and mRNA analyses indicated that BAT browning-related pathways might be activated. However, this finding lacks two critical lines of evidence: first, histological evidence for UCP1 expression, and second, morphological evidence of multilocular adipocytes.​To further strengthen the study findings, additional experiments—including Oil Red O staining or immunohistochemical staining for UCP1—will be conducted in future research.

## CONCLUSION

This study delved into the regulatory mechanism of the SP1/SREBP/UCP1 signaling pathway in an obese rat model and revealed a novel mechanism by which Erchen Decoction induces the browning tendency in white adipose tissue and inhibits fat synthesis. This finding not only enriches the scientific connotations of traditional Chinese medicine in treating obesity but also provides potential targets and strategies for developing new anti-obesity drugs based on the SP1/SREBP/UCP1 signaling pathway. Future research could further explore the precise regulatory mechanisms of specific components in Erchen Decoction on this signaling pathway and its application prospects in different types of obesity, providing more accurate and effective regimens for traditional Chinese medicine in treating obesity.

## Figures and Tables

**Fig. (1):**
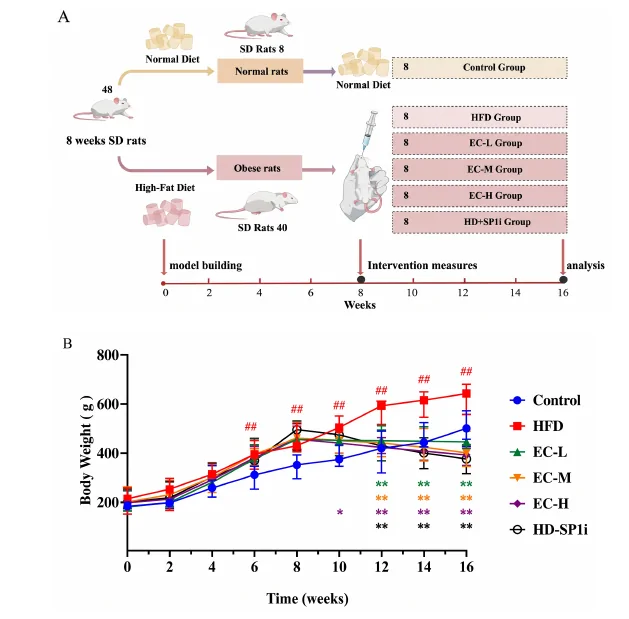
(**A**) Flow chart of SD rat obesity model preparation and gavage. (**B**) The effect of Erchen Decoction on the body mass of obese rats (n=8). Compared with the Control group, ^##^*P* < 0.01; compared with the HFD group, **P < 0.01.

**Fig. (2):**
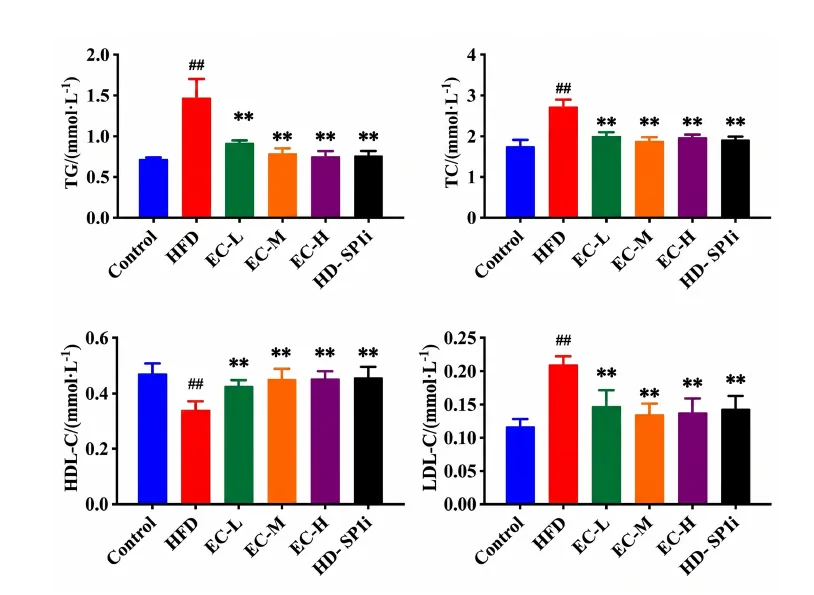
The effect of Erchen Decoction on the levels of total cholesterol (TC), triglyceride (TG), low-density lipoprotein (LDL-C), and high-density lipoprotein (HDL-C) in obese rats (n=8). Compared with the Control group, ^##^*P* < 0.01; compared with the HFD group, ​**P < 0.01.

**Fig. (3):**
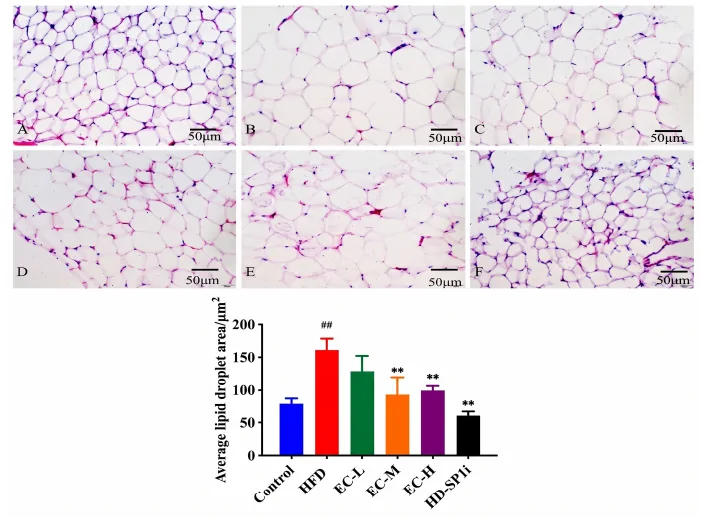
Observation of the morphology of rat adipose tissue by HE staining ×200 (n =3). (**A**) Control group; (**B**) HFD group; (**C**) Low-dose Erchen Decoction (EC-L) group; (**D**) Medium-dose Erchen Decoction (EC-M) group; (**E**) High-dose Erchen Decoction (EC-H) group; (**F**) High-dose + SP1 inhibitor (HD-SP1i) group. The bar chart represents the average lipid droplet area. Compared with the Control group, ^##^*P* < 0.01; compared with the HFD group, ​**P < 0.01.

**Fig. (4):**
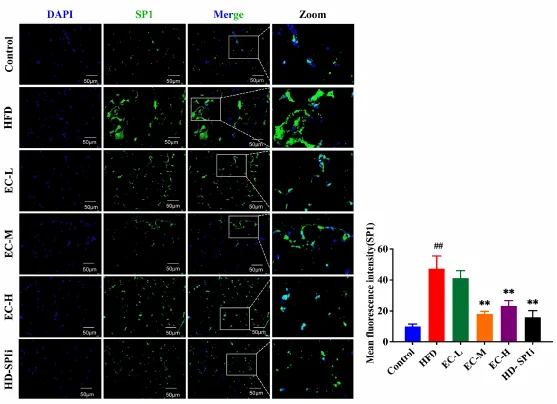
Immunofluorescence staining was used to observe the expression of SP1 (n=3). The bar chart shows the statistics of the average fluorescence intensity. Compared with the control group, ^##^*P*<0.01; compared with the HFD group, **P < 0.01.

**Fig. (5):**
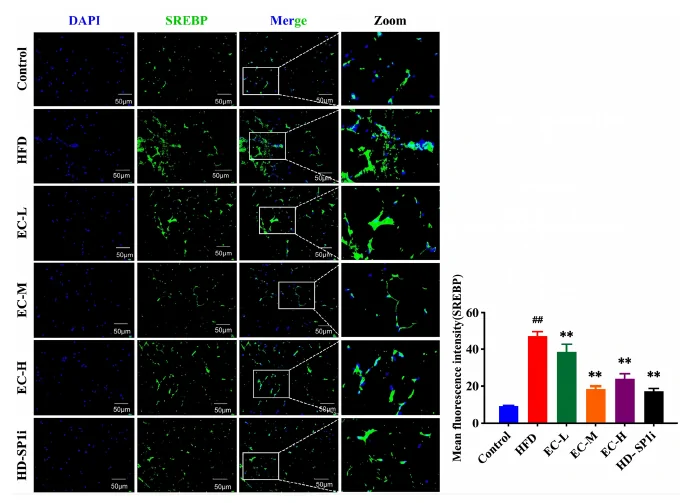
Immunofluorescence staining was used to observe the expression of SREBP (n=3). The bar chart shows the statistics of the average fluorescence intensity. Compared with the control group, ^##^*P* < 0.01; compared with the HFD group, **P < 0.01.

**Fig. (6):**
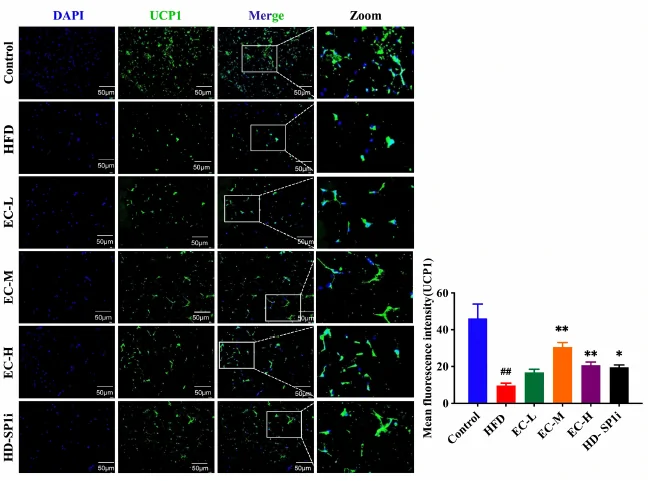
Immunofluorescence staining was used to observe the expression of UCP1 (n=3). The bar chart shows the statistics of the average fluorescence intensity. Compared with the control group, ^##^*P* < 0.01; compared with the HFD group,*P < 0.05, **P < 0.01.

**Fig. (7):**
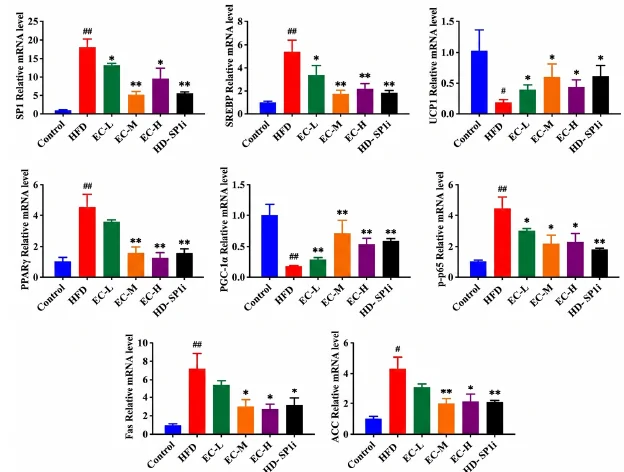
Effects of Erchen Decoction on the mRNA levels of SP1, SREBP, UCP1, PPARγ, PGC-1α, p-p65, Fas and ACC in adipose tissue (n=3). Compared with the Control group, ^#^*P* < 0.05, ^##^*P* < 0.01; compared with the HFD group, *P < 0.05, ​**P < 0.01.

**Fig. (8):**
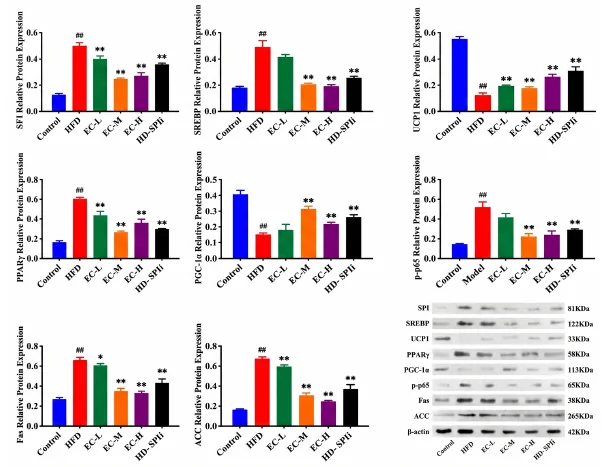
Protein expression levels of SP1, SREBP, UCP1, PPARγ, PGC-1α, p-p65, Fas, and ACC in obese rats (n=3). Compared with the Control group, ^##^*P* < 0.01; compared with the HFD group, *P < 0.05, ​**P < 0.01.

**Table 1:** Composition characteristics of ECD.

Latin Name	Chinese	Plant Part	Total Dosage (g)
*Pinelliae Rhizoma*	Banxia	Rhizome	665
*Citri Exocarpium Rubrum*	Jupi	Dry mature pericarp	665
*Poria*	Fuling	sclerotium	399
*Glycyrrhizae Radix et Rhizoma Praeparata cum Melle*	Zhigancao	Rhizome	200
*Zingiberis Rhizoma Recens*	Shengjiang	Rhizome	621
*Mume Fructus*	Wumei	Fructus	133

**Table 2:** PCR primer sequences.

Genes	Primer Name	Primer Sequence
SP1	SP1-F	*TCTTCCTCCTCTGGGGCTAC*
	SP1-R	*AGAGCTGGGAGTCAGGGTAG*
SREBP	SREBP-F	*AGAATGAGCACTGTGGCCTC*
	SREBP-R	*GCTTTTGTGAGCACTTCGCA*
UCP1	UCP1-F	*ATCAAACCCCGCTACACTGG*
	UCP1-R	*GCATAGGAGCCCAGCATAGG*
PPARγ	PPARγ-F	*CGCATTTTTCAAGGGTGCCA*
	PPARγ-R	*AGATCTCCTGGAGCAGAGGG*
PGC-1α	PGC-1α-F	*CTGGATGGCTTGGGACATGT*
	PGC-1α-R	*CTGTGGGTGTGGTTTGCATG*
p-p65	p-p65-F	*GAAAGCGCTTGAGCAACCAA*
	p-p65-R	*TCAGTCAGTCCGGTTTCAGC*
Fas	Fas-F	*AAGCTCCTTTGGCTGCTGAT*
	Fas-R	*TCATAGGTGGCAGGCTCTCT*
ACC	ACC-F	*CGGCAACAAACAAGGG*
	ACC-R	*CGTTACAACCAGGAAGCC*
GAPDH	GAPDH-F	*GAAGGTCGGTGTGAACGGAT*
	GAPDH-R	*AGTGATGGCATGGACTGTGG*

**Table 3:** Effect of ECD on biochemical parameters in HFD rats (x ± s, n=8).

Group	n	TC	TG	HDL-C	LDL-C
Control	8	1.758±0.149	0.725±0.018	0.471±0.037	0.118±0.011
HFD	8	2.721±0.180^##^	1.476±0.226^##^	0.342±0.029^##^	0.211±0.012^##^
EC-L	8	2.013±0.092**	0.922±0.027**	0.429±0.020**	0.148±0.024**
EC-M	8	1.885±0.100**	0.793±0.059**	0.454±0.036**	0.136±0.015**
EC-H	8	1.984±0.064**	0.753±0.066**	0.454±0.027**	0.138±0.021**
HD-SPli	8	1.923±0.076**	0.769±0.052**	0.459±0.038**	0.144±0.019**

**Note:** Compared with the Control group, ^##^*P* < 0.01; compared with the HFD group, **P < 0.01.

## Data Availability

The data and supportive information are available within the article.

## LIST OF ABBREVIATIONS

2-AAP: 2-acetylaminophenol
ACC: Acetyl-CoA Carboxylase
AKT: Protein Kinase B
ANOVA: Analysis of Variance
BAT: Brown Adipose Tissue
EC-H: High-dose Erchen Decoction
EC-L: Low-dose Erchen Decoction
EC-M: Medium-dose Erchen Decoction
FAS: Fatty Acid Synthase
FFAs: Free Fatty Acids
HD-SP1i: High-dose + SP1 Inhibitor
HDL-C: High-density Lipoprotein
HFD: High-Fat Diet
HSD: Honestly Significant Difference
HSL: Hormone-sensitive Lipase
IRS1: Insulin Receptor Substrate 1
LDL-C: Low-density Lipoprotein
PKA: Protein Kinase A
TC: Total Cholesterol
TCM: Traditional Chinese Medicine
TG: Triglyceride
UCP1: Uncoupling Protein 1
WAT: White Adipose Tissue
